# Supplementary material for: Areas of spatial overlap between common bottlenose dolphin, recreational boating, and small-scale fishery: management insights from modelling exercises
Source: PeerJ. 2023 Sep 28;11:e16111. doi: 10.7717/peerj.16111 (PMC10542390; doi:10.7717/peerj.16111)
Supplement: Supplemental Information 4 [file peerj-11-16111-s004.docx]

**Areas of spatial overlap between common bottlenose dolphin, recreational boating, and small-scale fishery: management insights from modelling exercises**

La Manna Gabriella^1,2,3^, Ronchetti Fabio^3^, Perretti Francesco^3^, Ceccherelli Giulia^1^

^1^ Università di Sassari, Dipartimento di Scienze Chimiche, Fisiche, Matematiche e Naturali, Sassari, Italy

^2^ National Biodiversity Future Center, Palermo, Italy

^3^ MareTerra Onlus, Environmental Research and Conservation, Sassari, Italy

**Supplementary materials**

Suppl. Mat. 1. Correlation matrix of the Pearson correlation coefficients (r) between the environmental predictors in the spring (a), summer (b) and (c) the whole period (spr-sum).

| a) spring | Aspect | Boats | Depth | Distance to coast | Distance to harbour | Fishnets | Traps | Chla | Seabed Habitat | Slope | SSTm | SSTr |
| --- | --- | --- | --- | --- | --- | --- | --- | --- | --- | --- | --- | --- |
| Aspect |  | -0.13 | 0.09 | -0.07 | 0.07 | 0.14 | -0.02 | 0.00 | 0.02 | 0.08 | 0.11 | 0.16 |
| Boats | -0.13 |  | 0.62 | -0.60 | -0.45 | 0.62 | 0.53 | 0.16 | 0.16 | 0.35 | -0.30 | 0.18 |
| Depth | 0.09 | 0.62 |  | **-0.84** | -0.55 | 0.62 | 0.46 | **0.83** | 0.26 | 0.19 | -0.22 | 0.26 |
| Distance to coast | -0.07 | -0.60 | -**0.84** |  | 0.34 | -0.52 | -0.35 | -0.46 | -0.18 | -0.31 | -0.08 | 0.06 |
| Distance to harbour | 0.07 | -0.45 | -0.55 | 0.34 |  | -0.45 | -0.76 | -0.48 | -0.20 | 0.08 | 0.77 | -0.28 |
| Fishnets | 0.14 | 0.62 | 0.62 | -0.52 | -0.45 |  | **0.83** | 0.22 | 0.44 | 0.16 | -0.22 | 0.31 |
| Traps | -0.02 | 0.53 | 0.46 | -0.35 | -0.76 | **0.83** |  | 0.32 | 0.21 | 0.21 | -0.65 | 0.11 |
| Chla | 0.00 | 0.16 | **0.83** | -0.46 | -0.48 | 0.22 | 0.32 |  | 0.18 | -0.01 | -0.24 | 0.03 |
| Seabed habitat | 0.02 | 0.16 | 0.26 | -0.18 | -0.20 | 0.44 | 0.21 | 0.18 |  | -0.04 | -0.05 | 0.16 |
| Slope | 0.08 | 0.35 | 0.19 | -0.31 | 0.08 | 0.16 | 0.21 | -0.01 | -0.04 |  | 0.13 | -0.12 |
| SSTm | 0.11 | -0.30 | -0.22 | -0.08 | 0.77 | -0.22 | -0.65 | -0.24 | -0.05 | 0.13 |  | -0.24 |
| SSTr | 0.16 | 0.18 | 0.26 | 0.06 | -0.28 | 0.31 | 0.11 | 0.03 | 0.16 | -0.12 | -0.24 |  |
| b) summer | Aspect | Boats | Depth | Distance to coast | Distance to harbour | Fishnets | Traps | Chla | Seabed Habitat | Slope | SSTm | SSTr |
| Aspect |  | -0.18 | 0.09 | -0.07 | 0.07 | 0.07 | 0.02 | 0.00 | 0.02 | 0.08 | -0.03 | 0.07 |
| Boats | -0.18 |  | 0.54 | -0.56 | -0.43 | 0.73 | 0.59 | 0.10 | 0.14 | 0.29 | -0.32 | 0.38 |
| Depth | 0.09 | 0.54 |  | **-0.84** | -0.55 | 0.46 | 0.44 | **0.86** | 0.26 | 0.19 | -0.44 | 0.32 |
| Distance to coast | -0.07 | -0.56 | **-0.84** |  | 0.34 | -0.46 | -0.46 | -0.50 | -0.18 | -0.31 | 0.05 | -0.67 |
| Distance to harbour | 0.07 | -0.43 | -0.55 | 0.34 |  | -0.37 | -0.59 | -0.49 | -0.20 | 0.08 | 0.77 | -0.09 |
| Fishnets | 0.07 | 0.73 | 0.46 | -0.46 | -0.37 |  | **0.84** | -0.05 | 0.19 | 0.22 | -0.34 | 0.34 |
| Traps | 0.02 | 0.59 | 0.44 | -0.46 | -0.59 | **0.84** |  | 0.20 | 0.18 | 0.28 | -0.37 | 0.43 |
| Chla | 0.00 | 0.10 | **0.86** | -0.50 | -0.49 | -0.05 | 0.20 |  | 0.24 | -0.02 | -0.32 | 0.06 |
| Seabed habitat | 0.02 | 0.14 | 0.26 | -0.18 | -0.20 | 0.19 | 0.18 | 0.24 |  | -0.04 | -0.18 | -0.05 |
| Slope | 0.08 | 0.29 | 0.19 | -0.31 | 0.08 | 0.22 | 0.28 | -0.02 | -0.04 |  | 0.13 | 0.27 |
| SSTm | -0.03 | -0.32 | -0.44 | 0.05 | 0.77 | -0.34 | -0.37 | -0.32 | -0.18 | 0.13 |  | 0.28 |
| SSTr | 0.07 | 0.38 | 0.32 | -0.67 | -0.09 | 0.34 | 0.43 | 0.06 | -0.05 | 0.27 | 0.28 |  |
| c) spr-sum | Aspect | Boats | Depth | Distance to coast | Distance to harbour | Fishnets | Traps | Chla | Seabed Habitat | Slope | SSTm | SSTr |
| Aspect |  | -0.16 | 0.09 | -0.07 | 0.07 | 0.13 | -0.01 | 0.00 | 0.02 | 0.08 | 0.00 | 0.20 |
| Boats | -0.16 |  | 0.58 | -0.58 | -0.45 | 0.75 | 0.61 | 0.11 | 0.15 | 0.28 | -0.35 | 0.35 |
| Depth | 0.09 | 0.58 |  | **-0.84** | -0.55 | 0.57 | 0.44 | **0.85** | 0.26 | 0.19 | -0.40 | 0.41 |
| Distance to coast | -0.07 | -0.58 | -0.84 |  | 0.34 | -0.53 | -0.41 | -0.48 | -0.18 | -0.31 | 0.02 | -0.22 |
| Distance to harbour | 0.07 | -0.45 | -0.55 | 0.34 |  | -0.44 | -0.69 | -0.49 | -0.20 | 0.08 | 0.79 | -0.33 |
| Fishnets | 0.13 | 0.75 | 0.57 | -0.53 | -0.44 |  | **0.87** | 0.07 | 0.31 | 0.20 | -0.37 | 0.49 |
| Traps | -0.01 | 0.61 | 0.44 | -0.41 | -0.69 | 0.57 |  | 0.25 | 0.16 | 0.27 | -0.49 | 0.11 |
| Chla | 0.00 | 0.11 | 0.65 | -0.48 | -0.49 | 0.07 | 0.25 |  | 0.21 | -0.02 | -0.32 | 0.04 |
| Seabed habitat | 0.02 | 0.15 | 0.26 | -0.18 | -0.20 | 0.31 | 0.16 | 0.21 |  | -0.04 | -0.15 | 0.15 |
| Slope | 0.08 | 0.28 | 0.19 | -0.31 | 0.08 | 0.20 | 0.27 | -0.02 | -0.04 |  | 0.14 | -0.01 |
| SSTm | 0.00 | -0.35 | -0.40 | 0.02 | 0.79 | -0.37 | -0.49 | -0.32 | -0.15 | 0.14 |  | -0.54 |
| SSTr | 0.20 | 0.35 | 0.41 | -0.22 | -0.33 | 0.49 | 0.11 | 0.04 | 0.15 | -0.01 | -0.54 |  |

Suppl. Mat. 2. Raster of the environmental variables used to model the likelihood of boat, fishing net, and dolphin presence (whole period): aspect; seafloor slope (degree); water depth (m); distance to the harbor (m); seabed habitat type; mean sea surface temperature (SSTm) and difference between the maximum and the minimum sea surface temperature (SSTr), averaged over the whole sampling period (spr-sum). Aspect was coded as follows: flat (0); N (1); NW (2); E (3); SE (4); S (5); SW (6); W (7); NE (8). Seabed habitat type was coded as follows: 1 = Mediterranean infralittoral rock (MB15); 2 = Coralligenous biocenosis (MC151); 3 = Mediterranean infralittoral coarse sediment (MB35); 4 = Mediterranean infralittoral sand (MB55); 5 = Biocenosis of Mediterranean muddy detritic bottoms (MC451); 6 = Mediterranean circalittoral coarse sediment (MC35); 7 = Biocenosis of Mediterranean open-sea detritic bottoms on shelf-edge (MD451); 8 = Biocenosis of *Posidonia oceanica* (MB252); 9= Facies of dead "mattes" of *Posidonia oceanica* without much epiflora (MB2523).

**
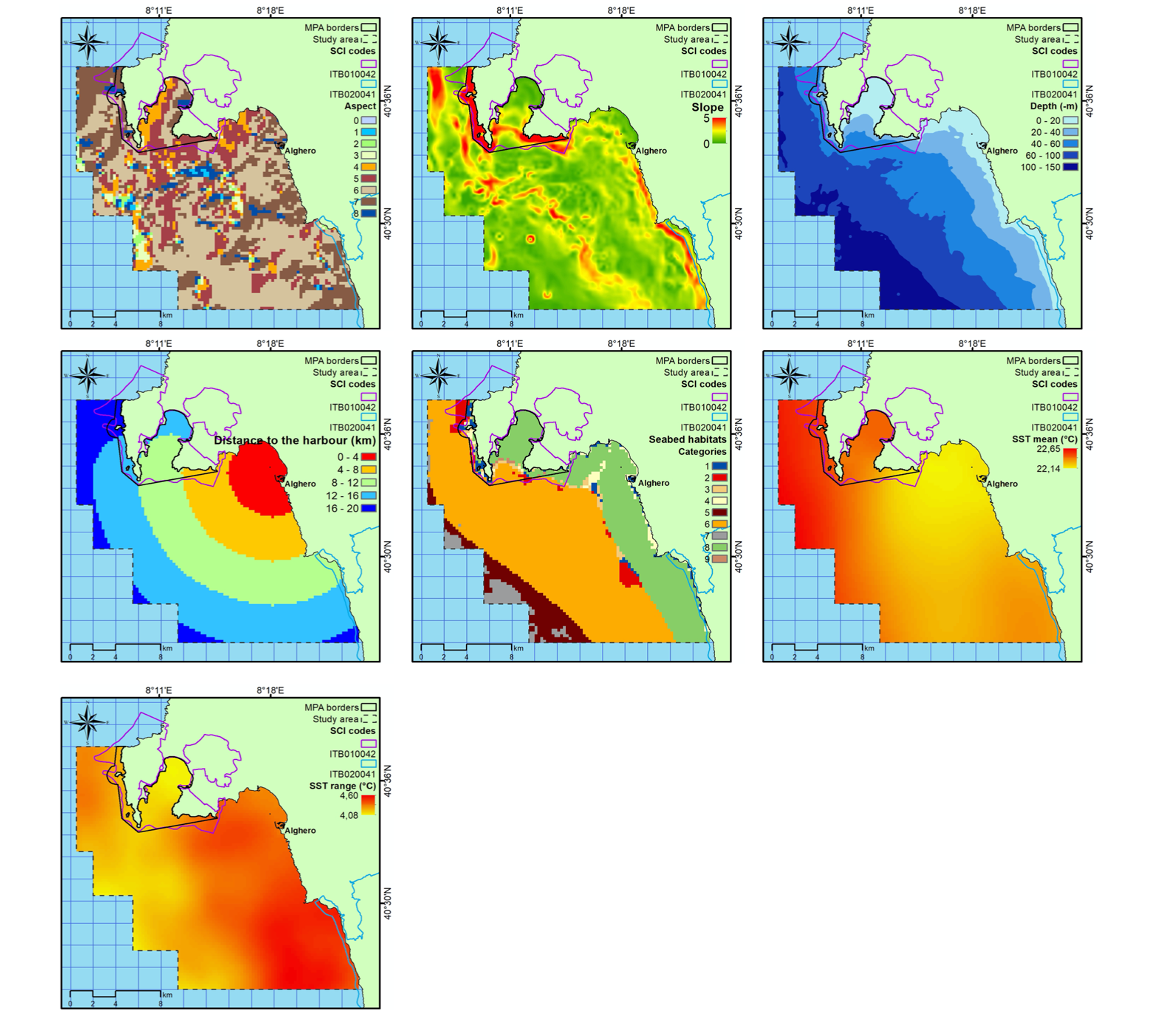
**

Suppl. Mat. 2. Effect of depth and aspect on the likelihood of boat presence (cloglog output) in spring (A), summer (B) and spr-sum (C).

**
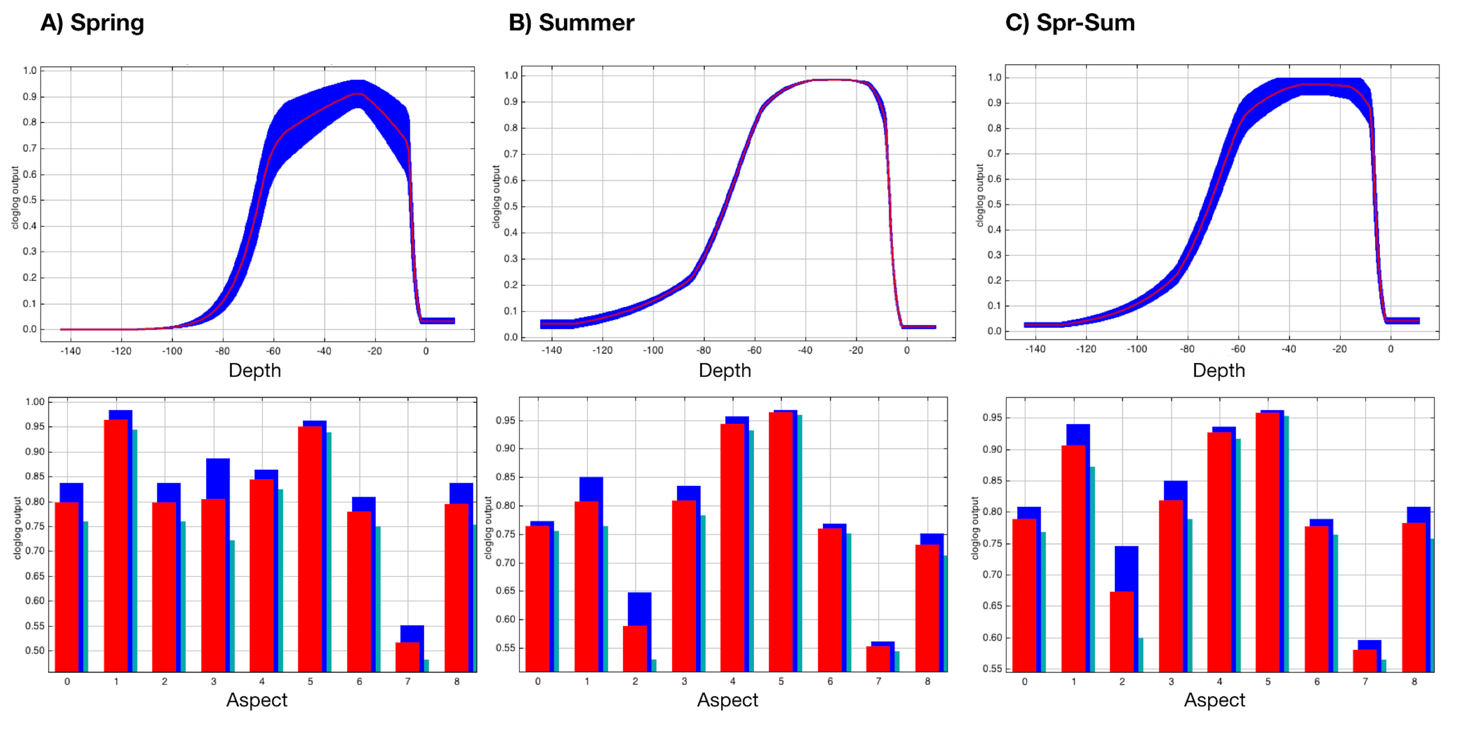
**

Suppl. Mat. 3. Effect of SSTm, SSTr, seabed habitat, distance to harbor and depth on the likelihood of fishing net presence (cloglog output) in spring (A), summer (B) and spr-sum (C).

**
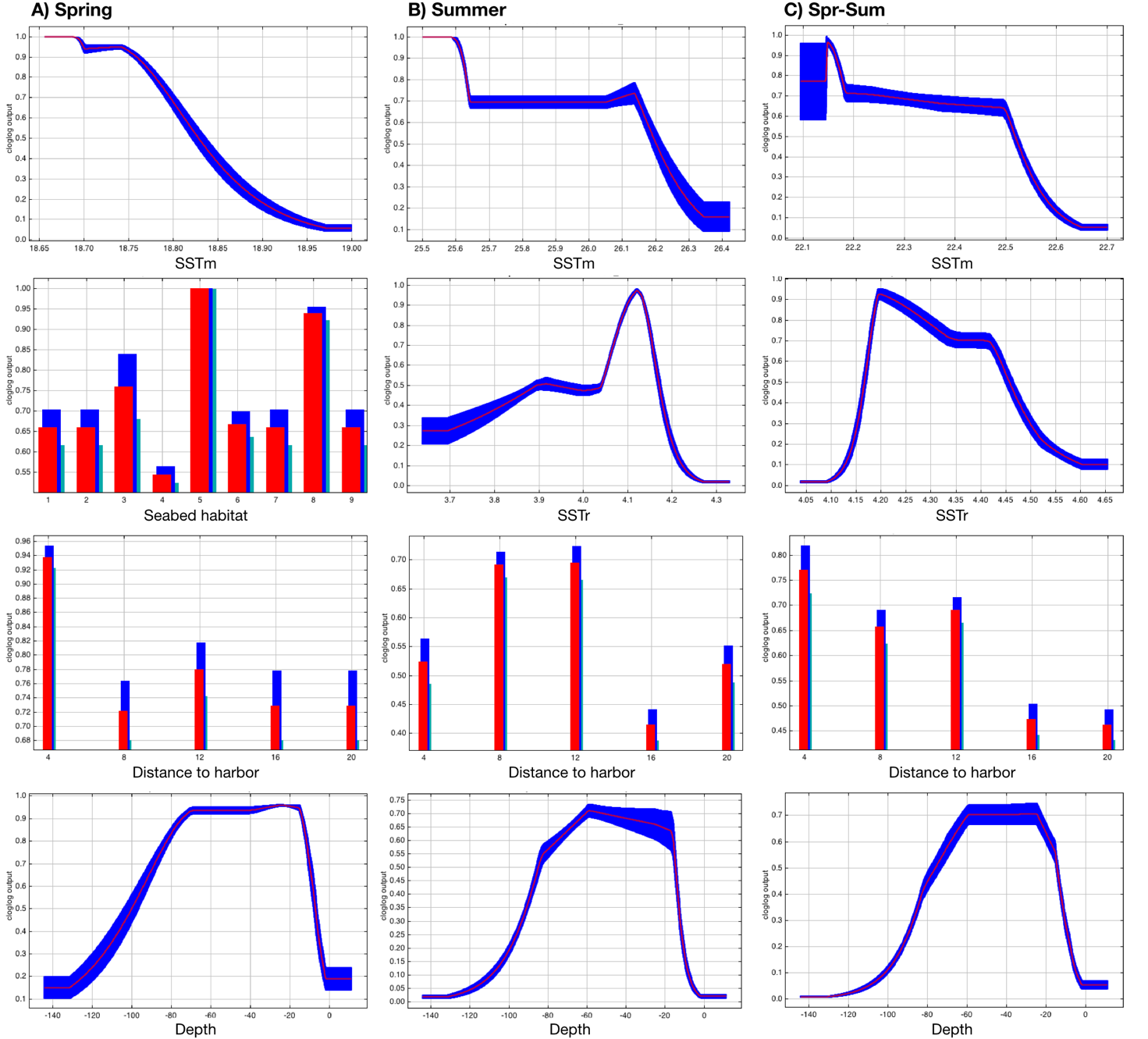
**
